# Mucosal-Associated Invariant T Cells Drive Bile Duct Inflammation

**DOI:** 10.1016/j.jcmgh.2026.101794

**Published:** 2026-04-29

**Authors:** Kathrine S. Nordhus, Fei Zheng, Natalie L. Berntsen, Oda Helgesen Ramberg, Laura Valestrand, Jonas Øgaard, Brian K. Chung, Xiaojun Jiang, Espen Melum

**Affiliations:** 1 Norwegian PSC Research Center, Department of Transplantation Medicine, Division of Surgery and Specialized Medicine, Oslo University Hospital, Oslo, Norway; 2 Research Institute of Internal Medicine, Division of Surgery and Specialized Medicine, Oslo University Hospital, Oslo, Norway; 3 Institute of Clinical Medicine, Faculty of Medicine, University of Oslo, Oslo, Norway; 4 Section of Gastroenterology, Department of Transplantation Medicine, Division of Surgery and Specialized Medicine, Oslo University Hospital, Oslo, Norway; 5 Hybrid Technology Hub-Centre of Excellence, Institute of Basic Medical Sciences, Faculty of Medicine, University of Oslo, Oslo, Norway

**Keywords:** Bile Duct Injection, Cholangitis, *E coli*, iVα19 Cα^−/−^ Tg Mice, MAIT Cells, *Mr1*^*+*^ B6-MAIT^CAST^ Mice, PSC, 5-OP-RU

## Abstract

**Background & Aims:**

Mucosal-associated invariant T cells recognize microbial-derived vitamin B metabolites presented by MHC I-related molecules. Because bile from patients with chronic biliary inflammation contain mucosal-associated invariant T antigens, we investigated whether intrabiliary mucosal-associated invariant T antigen exposure activates mucosal-associated invariant T cells and induce pathogenic biliary inflammation.

**Methods:**

*Escherichia coli*, phosphate-buffered saline, or the known mucosal-associated invariant T ligand 5-(2-oxopropylideneamino)-6-D-ribitylaminouracil were injected into bile ducts of mice with increased mucosal-associated invariant T cell frequencies (*Mr1*^*+*^ B6-MAIT^CAST^ and *i*Vα19 Cα^−/−^ Tg). Mice were monitored clinically and liver tissue analyzed after 1 to 14 days. Portal inflammation was assessed histologically. The immunophenotype of lymphocytes was determined by flow cytometry, and serum liver enzymes were measured. Hepatic mucosal-associated invariant T cell transcriptional profiles were analyzed by single-nucleus RNA sequencing.

**Results:**

Intrabiliary injection of *E coli* in *i*Vα19 Cα^−/−^ Tg mice caused cholangitis at day 2, elevated alanine aminotransferase, increased histologic grade of cholangitis, and portal accumulation of T-lymphocytes and macrophages. This coincided with marked hepatic mucosal-associated invariant T cell activation. Similar signs of experimental cholangitis were observed in mice containing 2% mucosal-associated invariant T cells (*Mr1*^*+*^ B6-MAIT^CAST^). Selective activation with 5-(2-oxopropylideneamino)-6-D-ribitylaminouracil in *i*Vα19 Cα^−/−^ Tg mice confirmed the mucosal-associated invariant T specificity of the inflammatory response. In *Mr1*^*+*^ B6-MAIT^CAST^ mice, the mucosal-associated invariant T cell frequency was too low to induce biliary inflammation. Single-nucleus RNA sequencing of mucosal-associated invariant T cells from 5-(2-oxopropylideneamino)-6-D-ribitylaminouracil–injected *i*Vα19 Cα^−/−^ Tg mice demonstrated antigen-driven transcriptional reprogramming toward a mucosal-associated invariant T–17-skewed phenotype with enrichment of T cell receptor signaling pathways.

**Conclusions:**

Mucosal-associated invariant T cells can drive pathogenic bile duct inflammation in vivo when locally exposed to antigens. These findings suggest that modulation of mucosal-associated invariant T–driven immune pathways may represent a therapeutic approach in inflammatory cholangiopathies.

## Graphical abstract

**Figure:**
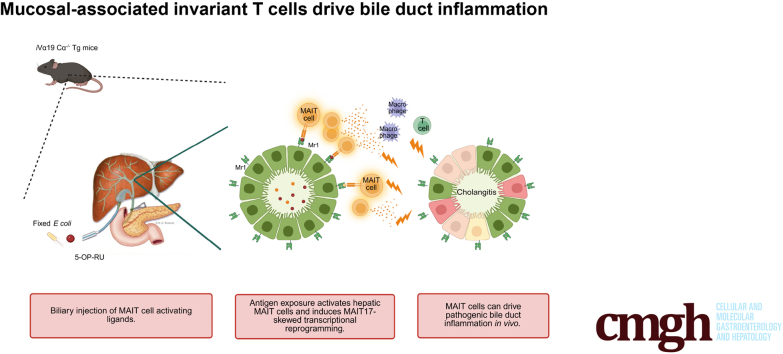

**What You Need to Know**

**Background**

Mucosal-associated invariant T cells recognize microbial metabolites presented by MHC I-related molecules. Their role in biliary inflammation remains unclear, despite detection of mucosal-associated invariant T cell antigens in bile from patients with chronic cholangiopathies.

**Impact**

We demonstrate that local antigen exposure activates hepatic mucosal-associated invariant T cells and drives bile duct inflammation. Transcriptomic analysis revealed proinflammatory reprogramming, identifying mucosal-associated invariant T cells as potential mediators of immune-mediated cholangiopathy.

**Future Directions**

Targeting MHC I-related molecule–dependent antigen presentation or mucosal-associated invariant T cell effector pathways may represent a therapeutic strategy in inflammatory cholangiopathies. Future studies should investigate chronic antigen exposure and translational relevance in human disease.

Chronic cholestatic liver diseases are characterized by destruction of the bile duct epithelium leading to subsequent cirrhosis, liver failure, and, potentially, cancer development.^1^ Primary sclerosing cholangitis (PSC), primary biliary cholangitis (PBC) and biliary atresia are clinically important cholangiopathies affecting adults and children.^2^ For PSC and biliary atresia, there are currently no United States Food and Drug Administration–/European Medicines Agency–approved effective medical therapies affecting disease course, whereas PBC has approved effective drugs, although they do not demonstrate effect in all patients.^3^ Therefore, cholangiopathies often lead to end-stage liver disease requiring liver transplantation, where cholangiopathies account for up to 20% to 25% of liver transplantations in adults and up to 80% in children.^4^^,^^5^ The current understanding of etiology and pathophysiology is limited to known predisposing genetic factors, whereas environmental contributors are largely unknown.^6^ Genetically, the cholangiopathies are complex diseases with multiple genetic contributors interacting with environmental factors, an observation that is further supported by functional studies.^7^^,^^8^ Lack of well-characterized experimental disease models complicates clarifications of how these genetic variants contribute to biliary immunopathology,^9^ and studies on specific contributing immune subsets are needed to identify potential treatment targets.

Unconventional T cells are a T cell subset that respond to nonpeptide antigens, in contrast to regular T cells that are activated by peptides.^10^ The 2 major subsets of unconventional T cells are mucosal associated invariant T (MAIT) and natural killer T (NKT) cells.^11^ For both MAIT and NKT cells, we have demonstrated a role in bile duct inflammation with the identification of antigens for both subsets in human bile^12^^,^^13^ and an activated state during experimental inflammation^14^^,^^15^ that is amenable to therapeutic manipulation.^14^ MAIT cells are the most abundant population of unconventional T cells in humans,^10^ with up to 5% of T cells in peripheral blood,^16^ and they are especially enriched in mucosal tissues^17^ and liver, with up to 45% of lymphocytes.^18^ Importantly, MAIT cells have also been shown to play an important role in pathogen defense and mucosal immunity.^19^, ^20^, ^21^ They are activated by metabolites of the riboflavin (vitamin B_2_) biosynthesis pathway that are produced by many commensal and pathogenic bacteria and presented on the highly conserved MHC I-related molecule (MR1).^17^ MAIT cells express a T cell receptor (TCR) composed of an invariant TCR α-chain (ie, Vα7-2-Jα33/Jα20/Jα12 in humans and Vα19-Jα33 in mice) together with a limited number of Vβ segments^17^^,^^22^ that are specific for a limited, but expanding array of antigens, with the pyrimidine 5-(2-oxopropylideneamino)-6-D-ribitylaminouracil (5-OP-RU) considered as a particularly potent antigen.^16^ Another interesting MR1 ligand in the context of biliary disease is the sulfated bile acid metabolite cholic acid 7-sulfate, which is the first endogenous antigen identified for MAIT cells in mice.^23^ Upon activation, MAIT cells secrete proinflammatory cytokines such as tumor necrosis factor α, interferon γ, granzyme B, interleukin (IL)-2, and IL-17. MAIT cells are characterized by high expression of cluster of differentiation (CD)161, IL-12 receptor, and IL-18 receptor and can be stimulated by IL-12/18 in a TCR-independent manner.^18^

Studies on MAIT cells in human cholangiopathies have so far been scarce, but it has been shown that they predominantly localize around bile ducts,^18^^,^^21^ and that patients with PSC have dysfunctional MAIT cells and a decreased frequency of circulating MAIT cells compared with non-PSC controls, similar to that found in patients with PBC or inflammatory bowel disease.^24^ Furthermore, it has been shown that biliary epithelial cells can present bacterial antigens via MR1 and activate MAIT cells in human liver.^21^ PSC is associated with altered microbiota of the gut and bile,^25^^,^^26^ and because MAIT cells are activated by microbial metabolites^17^ and bile from patients with PSC contains MR1-restricted antigens capable of activating MAIT cells,^12^ it is possible that bacterial metabolites stimulate immune-mediated damage to the bile ducts in PSC. MAIT cells can thus represent important modulators of microbial exposure in inflammatory bile duct diseases.^16^

In this study, we set out to determine if antigens locally presented in bile ducts could induce a MAIT cell–driven immune response that would be of relevance for the pathogenesis of inflammatory cholangiopathies. To that end, we challenged mice with increased levels of MAIT cells (*Mr1*^*+*^ B6- MAIT^CAST^, iVα19 Cα^−/−^ Tg mice) with intrabiliary injection of MAIT cell–activating antigens in formalin-fixed *Escherichia coli* and 5-OP-RU.

## Results

### Biliary Injection of Fixed *E coli* Causes Acute Biliary Immune Response

**Figure 1:**
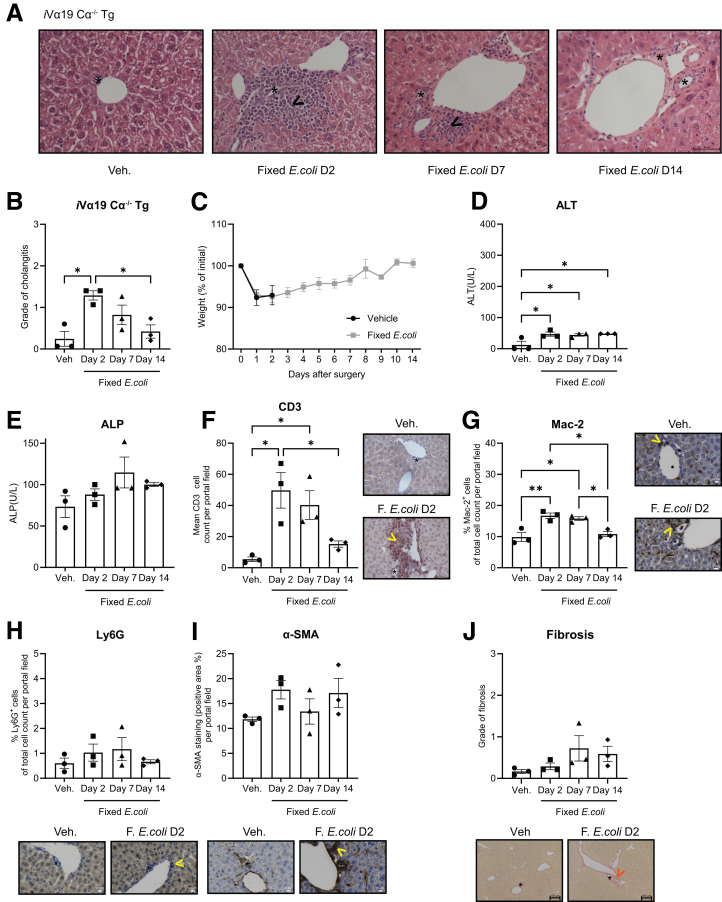
**Biliary injection of fixed *E coli* induces acute immune response in *i*Vα19 Cα^−/−^ Tg mice.** (*A*) Representative H&E staining (40×) of liver sections from *i*Vα19 Cα^−/−^ Tg mice 2, 7, and 14 days after biliary challenge with fixed *E coli* or vehicle (PBS). (*B*) Histologic evaluation of cholangitis in *i*Vα19 Cα^−/−^ Tg mice following biliary injection of fixed *E coli* or vehicle at the indicated days. Portal inflammation, necrosis and fibrosis were scored on a 0–3 scale: absent (0), mild (1), moderate (2), and severe (3). (*C*) Weight curve comparing biliary challenge of fixed *E coli* (n = 9 at day 2, n = 6 at day 7, and n = 3 at day 14) with vehicle (n = 3) at indicated days. (*D*) Serum values of ALT at days 2, 7, and 14 after surgery in indicated mice. (*E*) Serum values of ALP at days 2, 7, and 14 after surgery in indicated mice. (*F–I*) Representative immunohistochemical staining of the inflammatory infiltrate surrounding bile ducts (day 2) and corresponding quantification of (*F*) CD3^+^, (*G*) Mac2^+^, (*H*) Ly6G^+^, and (*I*) α-SMA^+^ cells at days 2, 7, and 14 after surgery in indicated mice. (*J*) Histologic evaluation of portal fibrosis in the indicated mice, scored as absent (0), mild (1), or moderate (2), with representative Sirius Red–stained liver sections (10×) from day 2, 7, and 14 after surgery. Scale bars represent 10 μm (Ly6G/Mac-2/α-SMA), 50 μm (H&E/CD3), and 100 μm (Sirius red); *asterisks*, bile ducts; *black arrowheads*, portal inflammation; *yellow arrowheads*, CD3/Ly6G/Mac-2– or α-SMA–positive cells; *orange arrowheads*, collagen depositions. Each symbol represents a single mouse. Representative results from 1 of 2 independent experiments are shown, presented as mean ± SEM. (*B, D,* and *H–J*) One-way ANOVA with Dunnett's adjustment for multiple comparisons; (*C*) 2-way repeated measures ANOVA with Šidák’s adjustment for multiple comparisons, and (*F* and *G*) 1-way ANOVA with Tukey’s adjustment for multiple comparisons tests were used to determine statistical differences: *∗P* < .05 and *∗∗P* < .01. ALP, alkaline phosphatase; SEM, standard error of the mean; Veh, vehicle.

To clarify if antigens potentially derived from the microbiota could induce a biliary immune response driven by MAIT cells, we challenged mice with intrabiliary injection of MAIT cell–activating antigens in fixed *E coli*.^27^ Because MAIT cells are rare in regular laboratory mouse strains,^28^ we used *Mr1*^*+*^ B6-MAIT^CAST^ mice that have an increased frequency of MAIT cells without any genetic manipulation^28^ and iVα19 Cα^−/−^ Tg mice, a MAIT^hi^ transgenic strain.^29^ When 5 × 10^10^ colony-forming unit (CFU)/mL fixed *E coli* were injected into iVα19 Cα^−/−^ Tg mice, we observed biliary inflammation (graded by portal inflammation, necrosis, and fibrosis) peaking at day 2 after surgery (Figure 1*A* and *B*) without any postoperative mortality. As expected, following major laparotomy, an initial weight loss was seen in all mice, irrespective of injected compound, with a complete regain of the starting weight by day 8 in the mice treated with fixed *E coli* (Figure 1*C*). Alanine transaminase (ALT) serum levels, an indicator of liver damage, were also significantly elevated in the *E coli*–treated group compared with vehicle controls at day 2 after surgery and remained higher than baseline throughout the experiment (Figure 1*D*). Alkaline phosphatase (Figure 1*E*) serum levels had a nonsignificant increase but remained within the reference range. The inflammatory infiltrate in mice injected with *E coli* was characterized by significantly increased accumulation of CD3-and galactose-specific soluble lectin 3 (Mac-2)–positive inflammatory cells (Figure 1*F* and *G*, respectively) at day 2 and day 7 after surgery, whereas lymphocyte antigen 6 complex locus G6D (Ly6G)^+^ cells were nearly absent at all investigated timepoints (Figure 1*H*). We further observed that biliary injection of fixed *E coli* led to mild fibrotic alterations in the liver with trends towards increased number of myofibroblasts around the bile ducts at day 2 (Figure 1*I*) and increased collagen depositions as visualized by staining with Sirius Red at day 7 and day 14 compared with vehicle controls (Figure 1*J*). The *E coli*–induced biliary inflammation gradually resolved with almost normal bile ducts by day 14 after surgery (Figure 1*A* and *B*).

**Figure 2:**
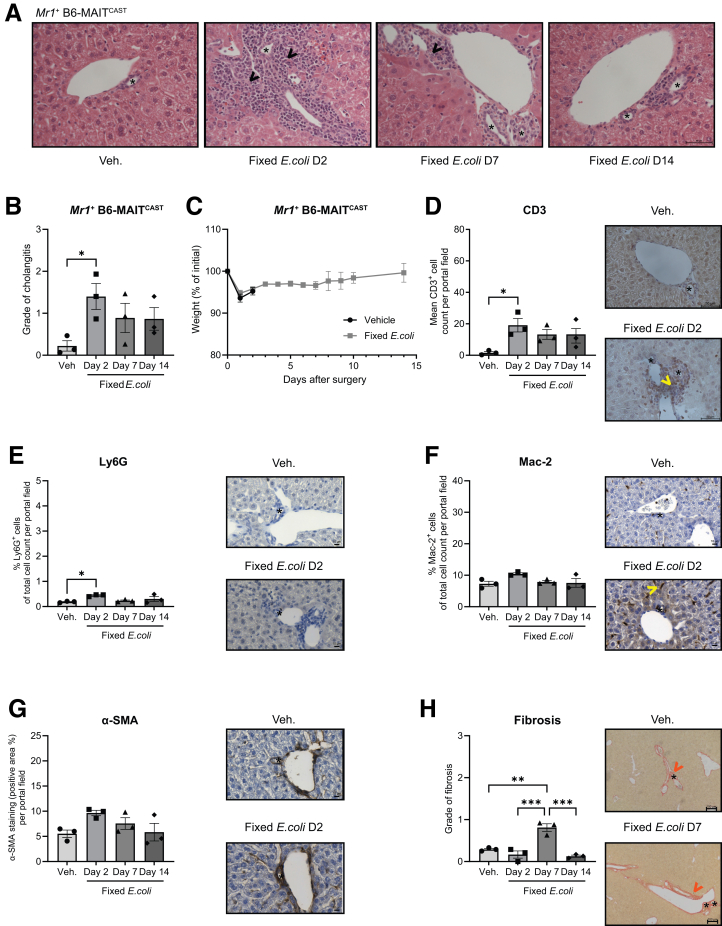
**Biliary injection of fixed *E coli* leads to inflammation of the portal area in *Mr1*^*+*^ B6-MAIT^CAST^ mice.** (*A*) Representative H&E staining (40×) of liver sections from *Mr1*^*+*^ B6-MAIT^CAST^ mice 2, 7, and 14 days after biliary challenge with fixed *E coli* or vehicle (PBS). (*B*) Histologic evaluation of cholangitis in *Mr1*^*+*^ B6-MAIT^CAST^ mice following biliary injection of fixed *E coli* or vehicle at the indicated days. Portal inflammation, necrosis, and fibrosis were scored on a 0–3 scale: absent (0), mild (1), moderate (2), and severe (3). (*C*) Weight curve comparing biliary challenge of fixed *E coli* (n = 9 at day 2, n = 6 at day 7, and n = 3 at day 14) with vehicle (n = 3) at indicated days in *Mr1*^*+*^ B6-MAIT^CAST^ mice. (*D–G*) Representative immunohistochemical staining of the inflammatory infiltrate surrounding bile ducts (day 2), and corresponding quantification of (*D*) CD3^+^, (*E*) Ly6G^+^, (*F*) Mac2^+^, and (*G*) α-SMA^+^ cells at days 2, 7, and 14 after surgery in indicated mice. (*H*) Histologic evaluation of portal fibrosis in the indicated mice, scored as absent (0), mild (1), or moderate (2), with representative Sirius Red–stained liver sections (10×) from day 2 (vehicle) and day 7 (*Ecoli*) after surgery. Scale bars represent 10 μm (Ly6G/Mac-2/α-SMA),50 μm (H&E/CD3), and 100 μm (Sirius red); *asterisks*, bile ducts; *black arrowheads*, portal inflammation; *yellow arrowheads*, CD3/Ly6G/Mac-2– or α-SMA–positive cells; *orange arrowheads*, collagen depositions. Each symbol represents a single mouse. Representative results from 1 of 2 independent experiments are shown, presented as mean ± SEM. (*B, E*, and *G*) One-way ANOVA with Dunnett’s adjustment for multiple comparisons; (*C*) 2-way repeated measures ANOVA with Šidák’s adjustment for multiple comparisons test; (*D, F*, and *H*) 1-way ANOVA with Tukey’s adjustment for multiple comparisons tests were used to determine statistical differences, ∗*P* < .05, ∗∗*P* < .01, and ∗∗∗*P* < .001. SEM, standard error of the mean; Veh, vehicle.

To determine if the MAIT-driven inflammation was only a feature of the iVα19 Cα^−/−^ Tg mouse, we performed intrabiliary challenge with 5 × 10^10^ CFU/mL fixed *E coli* in *Mr1*^*+*^ B6-MAIT^CAST^ mice, a mouse model without genetic alterations that have MAIT cells levels 20× higher than C57BL6/J mice,^28^ and observed a similar biliary immune response as in the iVα19 Cα^−/−^ Tg mice (Figure 2*A* and *B*). The portal inflammation in *Mr1*^*+*^ B6-MAIT^CAST^ mice was significantly higher in the mice receiving fixed *E coli* compared with vehicle at day 2 post surgery (Figure 2*B*). Despite this, the body weight loss (Figure 2*C*) was not affected compared with vehicle control. The inflammatory infiltrate was characterized by a significant recruitment of CD3- and Ly6G- but not Mac-2-positive inflammatory cells (Figure 2*D, E,* and *F*, respectively) at day 2 after surgery. Ongoing fibrogenesis was evidenced by a trend with elevated numbers of α-smooth muscle actin (α-SMA)^+^ cells at day 2 (Figure 2*G*) and significantly increased collagen depositions around the bile ducts at day 7 (Figure 2*H*).

### Fixed *E coli* Injection in the Bile Ducts Leads to Activation of Mucosal-Associated Invariant T Cells

**Figure 3:**
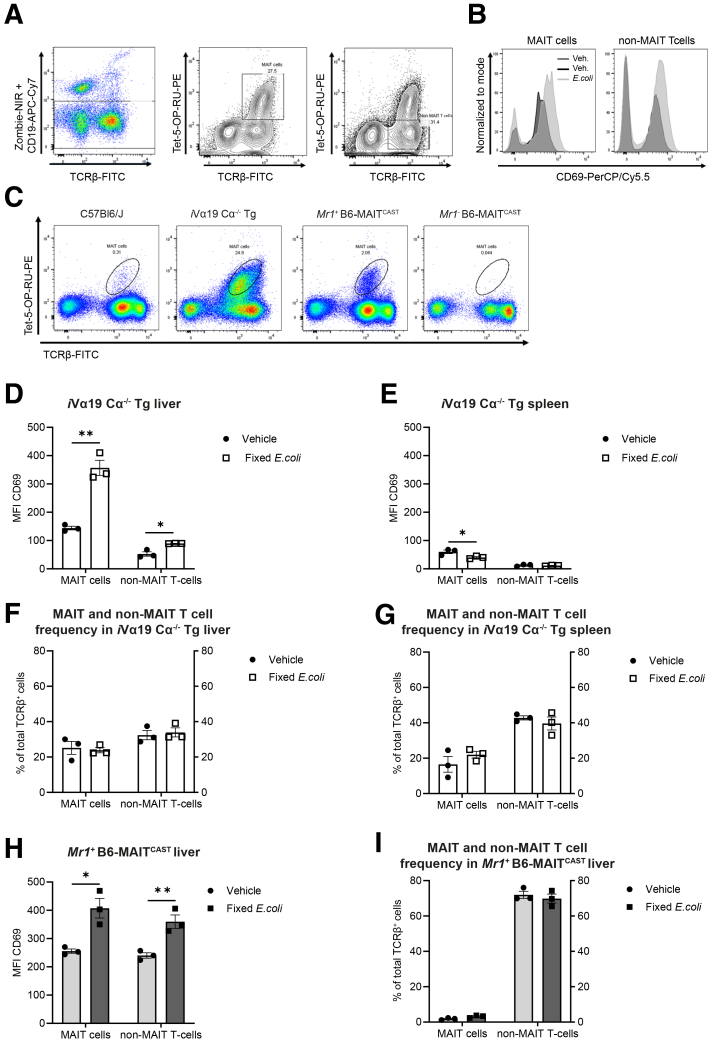
**Fixed *E coli* injection in the bile ducts leads to activation of MAIT cells.** Activation of hepatic and splenic MAIT cells after intrabiliary *E coli* injection as measured by flow cytometry. (*A*) Representative plots showing the gating strategies for MAIT and non-MAIT T cells in mononuclear cell suspension isolated from livers of *i*Vα19 Cα^−/−^ Tg mice 2 days after bile duct injection of fixed *E coli* or vehicle. (*B*) Representative histograms showing CD69 expression on MAIT and non-MAIT T cells. *Black* and *dark gray* histograms represent vehicle controls and the *light grey* histograms represent fixed *E coli-*treated mice. (*C*) Representative flow plots showing the frequency of hepatic MAIT cells in C57Bl6/J, *i*Vα19 Cα^−/−^ Tg, *Mr1*^*+*^ B6-MAIT^CAST^, and *Mr1*^*−*^ B6-MAIT^CAST^ mice. Bar graphs show CD69 expression measured by MFI on (*D*) hepatic and (*E*) splenic MAIT cells (tetramer^+^TCRβ^+^) cells and non-MAIT T cells (tetramer^−^TCRβ^+^) in *i*Vα19 Cα^−/−^ Tg mice injected with fixed *E coli* (n = 3) or vehicle (n = 3). Numbers indicate the frequency of MAIT and non-MAIT T cells in (*F*) liver and (*G*) spleen. (*H*) Bar graphs show CD69 expression measured by MFI of hepatic MAIT and non-MAIT T cells in *Mr1*^*+*^ B6-MAIT^CAST^ mice injected with fixed *E coli* (n = 3) or vehicle (n = 3). (*I*) Numbers indicate the frequency of hepatic MAIT and non-MAIT T cells in *Mr1*^*+*^ B6-MAIT^CAST^ mice. Representative results from 1 of 2 independent experiments are shown, presented as mean ± SEM. (*D–I*) Two-tailed Student’s *t* test was used to determine statistical differences; *∗P* < .05*,* and *∗∗P* < .01. MFI, median fluorescence intensity; SEM, standard error of the mean.

**Figure 4:**
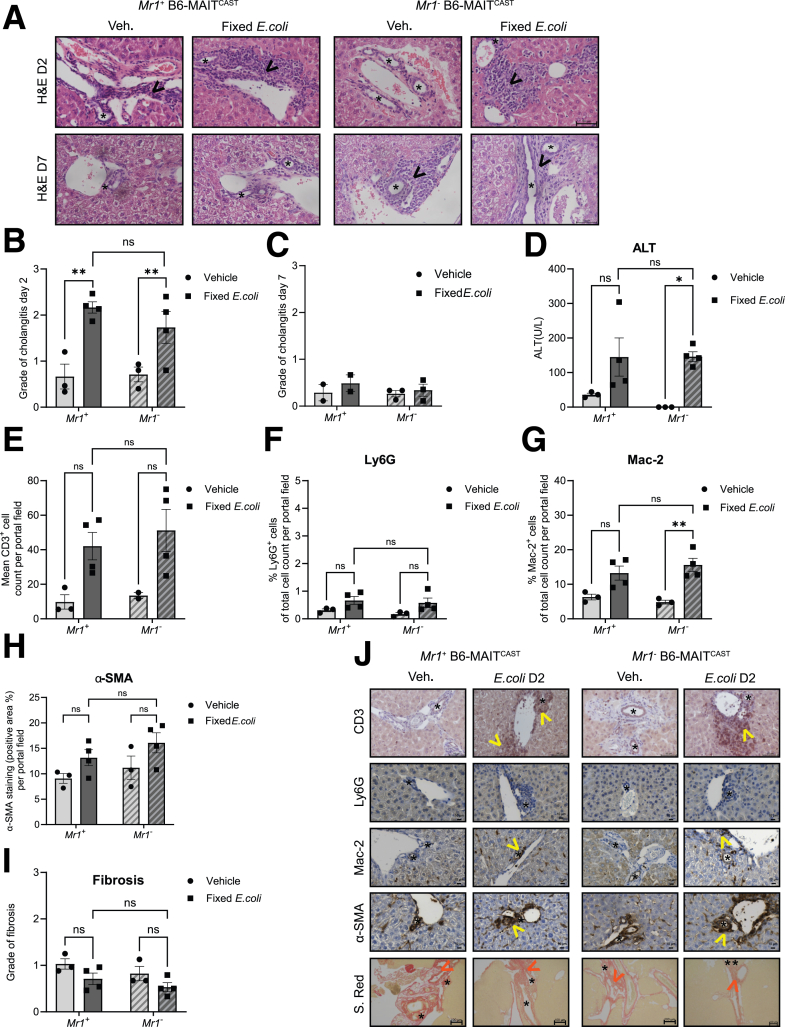
***Mr1*^*−*^ B6-MAIT^CAST^ mice biliary injected with fixed *E coli* develop cholangitis.** (*A*) Representative H&E staining (40×) of liver sections from *Mr1*^*+*^ B6-MAIT^CAST^ and *Mr1*^*−*^ B6-MAIT^CAST^ mice comparing biliary injection of fixed *E coli* with vehicle after 2 and 7 days. (*B* and *C*) Histologic evaluation of cholangitis in *Mr1*^*+*^ B6-MAIT^CAST^ and *Mr1*^*−*^ B6-MAIT^CAST^ mice following biliary injection of fixed *E. coli* or vehicle at (*B*) day 2 and (*C*) day 7. Portal inflammation, necrosis and fibrosis were scored on a 0–3 scale: absent (0), mild (1), moderate (2), and severe (3) (*D*) Serum values of ALT at day 2 after surgery in indicated mice. (*E–H*) Quantitative analysis of inflammatory infiltrates surrounding bile ducts, showing (*E*) mean CD3⁺ cell count, (*F*) Ly6G⁺ cells (% of total cell count), (*G*) Mac2⁺ cells (% of total cell count), and (*H*) α-SMA⁺ staining (% positive area) per portal field at day 2 after surgery in indicated mice. (*I*) Histologic evaluation of portal fibrosis in the indicated mice, scored as absent (0), mild (1), or moderate (2) at day 2 after surgery. (*J*) Representative immunohistochemical staining of the inflammatory infiltrate (CD3^+^, Ly6G^+^, Mac-2^+^, and α-SMA^+^ cells) (40×) and portal fibrosis (Sirius Red staining) (10×) surrounding bile ducts. Scale bars represent 10 μm (Ly6G/Mac-2/α-SMA), 50 μm (CD3 and H&E), and 100 μm (Sirius Red); *asterisks*, bile ducts; *black arrowheads*, portal inflammation; *yellow arrowheads*, CD3/Ly6G/Mac-2– or α-SMA–positive cells; *orange arrowheads*, collagen depositions. Each symbol represents a single mouse. Representative results from 1 independent experiment is shown, presented as mean ± SEM. (*B–F* and *I*) Two-way ANOVA with Šidák’s adjustment for multiple comparisons test and (*G* and *H*) 2-way ANOVA with Tukey’s adjustment for multiple comparisons tests were used to determine statistical differences, *∗P* < .05 and *∗∗P* < .01. *Mr1*^*+*^, *Mr1*^*+*^ B6-MAIT^CAST^; *Mr1*^−^, *Mr1*^*−*^ B6-MAIT^CAST^; SEM, standard error of the mean; Veh, vehicle.

After having demonstrated that fixed *E coli* could induce biliary inflammation in mice with increased levels of MAIT cells, we aimed to determine the contribution of MAIT cells compared with other T cells. To that end, we isolated hepatic and splenic lymphocytes from iVα19 Cα^−/−^ Tg mice 2 days after intrabiliary injection with either vehicle or fixed *E coli* and subjected the samples to flow cytometry analysis (gating strategy shown in Figure 3*A–C*). Hepatic MAIT cells had a clear and increased expression of the activation marker CD69 (Figure 3*D*), whereas non-MAIT T cells (gating definition: TCRβ^+^, MR1-Tet^−^) only had a minor increase in CD69 expression (Figure 3*D*) when comparing mice receiving fixed *E coli* with vehicle. A slight significant increase in the activation status of splenic MAIT cells was observed, suggesting some systemic effect of the injection (Figure 3*E*). Following *E coli* injection, the MAIT cell fraction was similar as in mice receiving vehicle (Figure 3*F* and *G*). Next, we performed the same experiment in *Mr1*^*+*^ B6-MAIT^CAST^ mice, which have one-tenth of the number of MAIT cells compared with iVα19 Cα^−/−^ Tg mice. In this model, we also observed a clear activation of hepatic MAIT cells with significantly increased expression of activation marker CD69 (Figure 3*H*). As expected, given the lower number of MAIT cells, non-MAIT T cells were more clearly activated in this model, with significantly increased CD69 expression also in the non-MAIT T cell fraction (Figure 3*H*). Also, in this model, we did not observe any differences in the MAIT cell fraction of lymphocytes (Figure 3*I*). The lower number of MAIT cells in the spleen in these mice did not allow for examination of activation status. To investigate if the biliary inflammation induced by non-MAIT T cells was secondary to MAIT–driven cholangitis, we subjected MAIT cell–deficient mice (*Mr1*^*−*^ B6-MAIT^CAST^) and *Mr1*^*+*^ B6-MAIT^CAST^ mice to intrabiliary fixed *E coli* or vehicle injection. A mild biliary immune response with an increased cholangitis score was observed at day 2 after surgery in both groups (*Mr1*^*+*^*: E coli*: 2.17 vs vehicle: 0.67; *P* = .0037 and *Mr1*^*−*^*: E coli*: 1.73 vs vehicle: 0.71; *P* = .034) (Figure 4*A* and *B*). By day 7 following surgery, the grade of cholangitis was equally reduced in both groups, irrespective of injected substance (Figure 4*C*). ALT values (Figure 4*D*), along with immunohistology examination of the inflammatory infiltrate and fibrosis formation (Figure 4*E–J*), demonstrated a consistent immune response and comparable outcomes between the genotypes, thereby indicating that fixed *E coli* possibly contains other antigens recognized by another immune subset(s) capable of inducing biliary inflammation.

### Selective Activation of Hepatic Mucosal-Associated Invariant T Cells by 5-(2-oxopropylideneamino)-6-D-ribitylaminouracil

**Figure 5:**
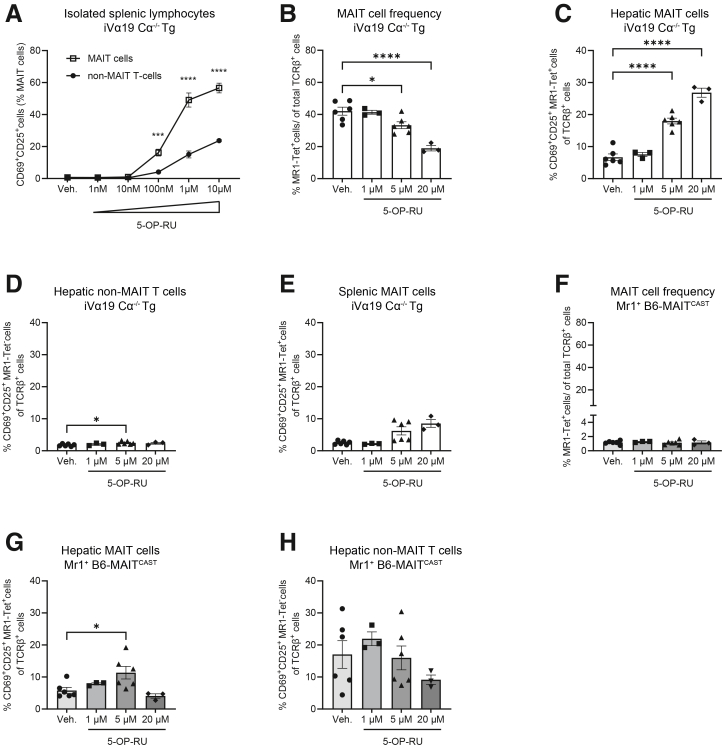
**In vitro stimulation of MAIT cells with 5-OP-RU and activation of hepatic MAIT cells after intrabiliary 5-OP-RU injection as measured by flow cytometry.** (*A*) Vehicle or increasing concentrations of 5-OP-RU (1 nM, 10 nM, 100 nM, 1 μM, 10 μM) were added to isolated splenic lymphocytes from iVα19 Cα^−/−^ Tg mice as indicated. Cells were incubated overnight and subsequently analyzed by flow cytometry. The representative curve graph shows the frequency of activated CD69^+^CD25^+^ MAIT and non-MAIT T cells in vitro. (*B*) Bar graphs shows the frequency of hepatic MAIT cells defined as tetramer^+^TCRβ^+^ cells in *i*Vα19 Cα^−/−^ Tg mice 1 day after biliary injection with 5-OP-RU (1 μM, 5 μM, 20 μM) (n = 3, n = 6, and n = 3, respectively) or vehicle (n = 6) and percentage of CD69^+^CD25^+^ hepatic (*C*) MAIT cells, (*D*) non-MAIT T cells (defined as tetramer^−^TCRβ^+^ cells), and (*E*) splenic MAIT cells. (*F*) Bar graphs show the frequency of hepatic MAIT cells in *MR1*^*+*^ B6-MAIT^CAST^ mice following biliary injection with 5-OP-RU (1 μM, 5 μM, 20 μM) (n = 3, n = 6, and n = 3, respectively) or vehicle (n = 6) 1 day after injection, and percentage of CD69^+^CD25^+^ hepatic (*G*) MAIT cells and (*H*) non-MAIT T cells. Each symbol represents a single mouse. (*A*) Representative results from 1 of 2 independent experiments are shown, presented as mean ± SEM of 3 replicates. (*B–H*). Combined results from 2 separate experiments (Round 1: Veh, 1 μM, 5 μM 5-OP-RU and Round 2: Veh, 5 μM, and 20 μM) are shown, presented as mean ± SEM. (*A*) Two-way ANOVA with Šidák`s adjustment for multiple comparisons; (*B–D, F*, and *G*) 1-way ANOVA with Dunnett’s adjustment for multiple comparisons; and (*E* and *H*) Brown-Forsythe and Welch’s ANOVA tests were used to determine statistical differences, *∗P* < .05, *∗∗∗P* < .001, and *∗∗∗∗P* < .0001. MFI, median fluorescence intensity; Veh, vehicle.

Because some activation of non-MAIT T cells was observed following biliary injection of fixed *E coli* in iVα19 Cα^−/−^ Tg mice, we selectively activated MAIT cells using 5-OP-RU injection to clarify the contribution of MAIT cells vs non-MAIT T cells. The potency and specificity of 5-OP-RU was first tested on isolated splenic lymphocytes from iVα19 Cα^−/−^ Tg mice ex vivo, and MAIT and non-MAIT T cell abundance and activation status was quantified by flow cytometry (Figure 5*A*). When iVα19 Cα^−/−^ Tg mice received intrabiliary 5-OP-RU injection, the hepatic MAIT cell frequency was reduced in a dose-dependent manner, likely due to downregulation of TCR following activation^30^ (Figure 5*B*) as a simultaneous dose-dependent increase in proportion of CD69^+^CD25^+^ MAIT cells was observed (Figure 5*C*). In contrast, hepatic non-MAIT T cells only had a minor increase in CD69^+^CD25^+^ expression following injection of 5 μmol/L 5-OP-RU (Figure 5*D*). The activation status of splenic MAIT cells was not significantly affected by different doses, underscoring the bile duct specificity of the disease model (Figure 5*E*).

When *Mr1*^*+*^ B6-MAIT^CAST^ mice received intrabiliary injection with increasing doses of 5-OP-RU, the hepatic MAIT cell frequency surprisingly remained unchanged between the treatment groups (Figure 5*F*). We did, however, observe an increase in the proportion of CD69^+^CD25^+^ MAIT cells, with a peak at 5 μmol/L 5-OP-RU (5 μmol/L 5-OP-RU: 11% vs vehicle: 6%; *P* = .021) (Figure 5*G*), but with no differences between vehicle-injected mice and mice injected with 20 μmol/L 5-OP-RU (Figure 5*G*). This could be due to a combination of effects, where activated MAIT cells become exhausted and downregulate their TCRs, as we observed for the iVα19 Cα^−/−^ Tg mice following a powerful response to a high dose of 5-OP-RU, and the harvesting timepoint was too late to capture it, especially because the MAIT cell frequency is only 2% in this mouse strain. The activation status of hepatic non-MAIT T cells was not significantly different between treatment groups (Figure 5*H*), and we did not investigate the activation status of peripheral splenic MAIT cells due to the low number in this model.

**Figure 6:**
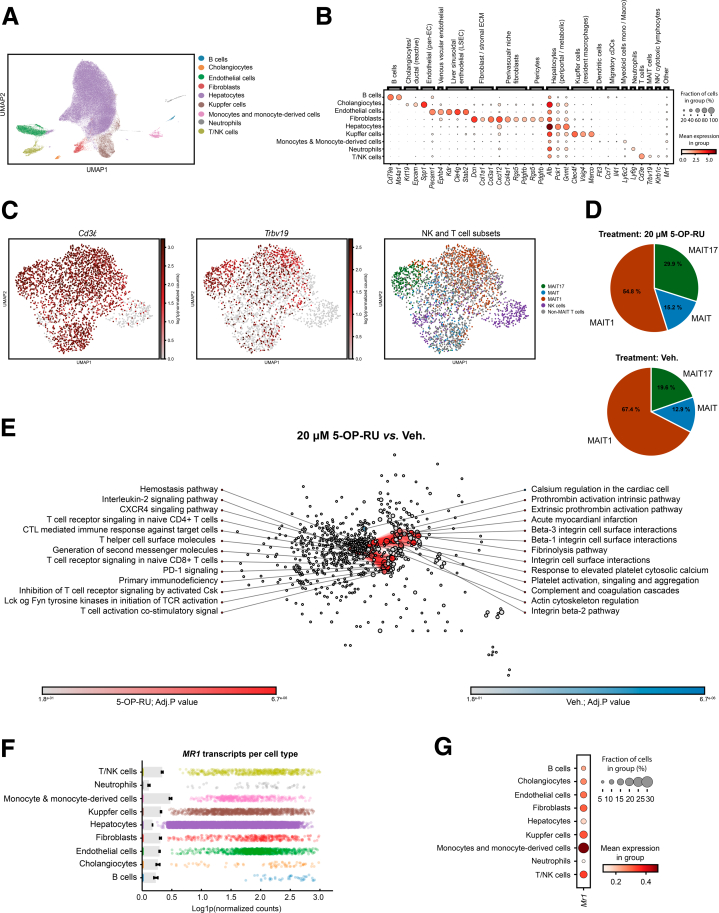
**snRNA-seq demonstrates expansion and activation of MAIT cells following biliary injection of 5-OP-RU.** (*A*) UMAP projection of snRNA-seq data showing major parenchymal and nonparenchymal cell populations, including hepatocytes, B cells, cholangiocytes, endothelial cells, fibroblasts, Kupffer cells, monocyte and monocyte-derived cells, neutrophils, and T/NK cells. (*B*) Dot plot displaying canonical gene expression markers used to define each cell population. Dot size indicates the fraction of cells expressing each gene, and color intensity reflects mean expression levels. (*C*) UMAPs showing expression of *Cd3ε* (*left panel*) and *Trbv19* (*middle panel*) within the T/NK cluster and visualization of T cell subclusters (*right panel*) identifying MAIT17 cells, MAIT1 cells, MAIT cells, NK cells, and non-MAIT T cells, (log1p(normalized counts)). (*D*) Pie charts with proportional representation of MAIT, MAIT1, and MAIT17 cells following biliary injection with 5-OP-RU or vehicle control. (*E*) Pathway enrichment analysis created using the Enrichr^31^ web service and the Bioplanet 2019^32^ reference database web service showing the significantly upregulated pathways visualized by the top 25 regulated terms in the 5-OP-RU– (*red dots*) and vehicle-treated (*blue*) mice (of a total of 56 and 5 significantly regulated terms after Benjamini-Hochberg correction, respectively), grouped by gene similarity (pathways sharing genes are closer to each other). (*F*) *Mr1* expression per hepatic cell type in *i*Vα19 Cα^−/−^ Tg mice, log1p(mean normalized counts ± SEM). (*G*) Dot plot demonstrating the fraction of cells in each cell type cluster expressing *Mr1* and the mean *Mr1* expression level (log1p normalized counts, color intensity) in each cluster. SEM, standard error of the mean; Trbv19, T cell receptor beta variable 19; UMAP, Uniform Manifold Approximation and Projection.

After having established that intrabiliary injection of 5-OP-RU activated hepatic MAIT cells, we comprehensively characterized the transcriptional response using single-nucleus RNA sequencing (snRNA-seq) of livers from *i*Vα19 Cα^−/−^ Tg mice receiving either 20 μmol/L 5-OP-RU (n = 3) or vehicle controls (n = 3). All major parenchymal and nonparenchymal cell populations were identified (Figure 6*A* and *B*), and within the T/NK cell cluster, we defined MAIT cells as *Cd3e*^*+*^, *Trbv19*^*+*^ cells that comprised 30% of this cluster. In the T/NK cell cluster, MAIT1 and MAIT17 cells were transcriptionally distinct from NK cells, but partially overlapped with conventional T cells, reflecting shared lineage programs^33^ (Figure 6*C*). Cells from mice receiving 5-OP-RU had a skewing towards MAIT17, whereas MAIT1 cells were relatively reduced (Figure 6*D*). Differential expression analysis identified 98 significantly upregulated genes in MAIT cells from 5-OP-RU–treated mice (Supplementary Table 1) with components of T cell receptor complex (*Cd3ε* and *Trbv19*; adjusted *P* < .01), transcription factors associated with MAIT cell identity ([*Rorc*; adjusted *P* = .017] and [*Zbtb16*; adjusted *P* < .01]), the tissue-residency marker (*Itgae*; adjusted *P* = .05), and chemokine receptor (*Cxcr6*; adjusted *P* < .01). In contrast, established markers for MAIT1 phenotype were either downregulated (*Gzmb*; adjusted *P* < .01) or not differentially expressed (*Tbx21*; adjusted *P* = .18), compared with vehicle control. By using a Pathway enrichment analysis, we observed activation of TCR signaling, costimulatory pathways, cytokine signaling, and effector immune programs, consistent with antigen-driven activation of the MAIT cells (Figure 6*E*). In contrast, MAIT cells from vehicle-treated mice exhibited an enrichment profile reflecting maintenance-associated transcriptional program during steady state.^34^ In the non-MAIT T cell population, we detected 34 enriched pathways, where only 2 of the pathways were related to T cell activation, most likely due to bystander activation and secondary to MAIT cell activation. The snRNA-seq dataset also allowed examination of *Mr1* transcripts in various cell types. Monocyte-derived cells and Kuppfer cells represented the main source of *Mr1* transcripts, whereas cholangiocytes displayed detectable levels comparable to or exceeding those observed in B cells (Figure 6*F* and *G*). These levels are in line with what we could observe in an independent dataset^35^ and indicate that cholangiocytes could act as local antigen-presenting cells (APCs) shaping the MAIT–driven immune response.

**Figure 7:**
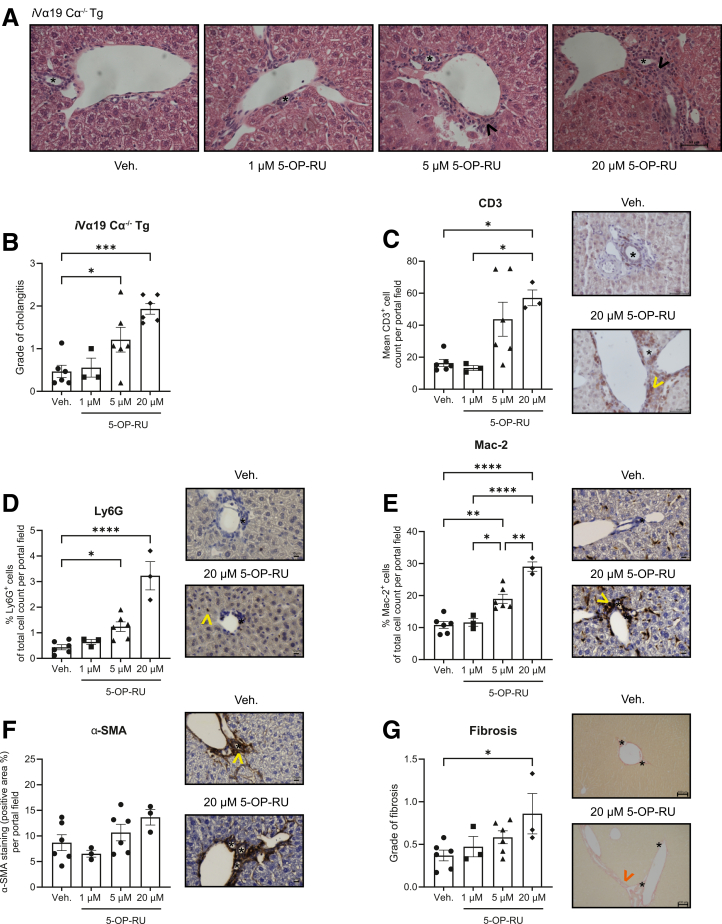
**Direct activation of MAIT cells by 5-OP-RU leads to cholangitis.** (*A*) Representative H&E staining (40×) of liver sections from iVα19 Cα^−/−^ Tg mice comparing biliary injection of increasing concentrations of 5-OP-RU with vehicle at day 1 after injection. (*B*) Histologic evaluation of cholangitis in *i*Vα19 Cα^−/−^ Tg mice following biliary injection of increasing concentrations of 5-OP-RU (n = 3–6) or vehicle (n = 6) at day 1 post surgery. Portal inflammation, necrosis and fibrosis were scored on a 0-3 scale: absent (0), mild (1), moderate (2), and severe (3). (*C–F*) Representative immunohistochemical staining of the inflammatory infiltrate surrounding bile ducts and corresponding quantification of (*C*) CD3^+^, (*D*) Ly6G^+^, (*E*) Mac2^+^, and (*F*) α-SMA^+^ cells at day 1 after surgery in indicated mice. (*G*) Histologic evaluation of portal fibrosis in the indicated mice, scored as absent (0), mild (1), or moderate (2), with representative Sirius Red–stained liver sections (10×) from day 1 after injection of vehicle in indicate mice. Scale bars represent 10 μm (Ly6G/Mac-2/α-SMA), 50 μm (CD3/H&E), and 100 μm (Sirius Red); *asterisks*, bile ducts; *black arrowheads*, portal inflammation; *yellow arrowheads*, CD3/Ly6G/Mac-2– or α-SMA–positive cells; *orange arrowheads*, collagen depositions. Each symbol represents a single mouse. Representative results from 1 of 2 independent experiments are shown, presented as mean ± SEM. (*B, D, F*, and *G*) One-way ANOVA with Dunnett’s adjustment for multiple comparisons; (*C*) Brown-Forsythe ANOVA test with Dunnett’s adjustment for multiple comparisons; and (*E*) 1-way ANOVA with Tukey’s adjustment for multiple comparisons tests were used to determine statistical differences; *∗P* < .05, *∗∗P* < .01, *∗∗∗P* < .001, and *∗∗∗∗P* < .0001. SEM, standard error of the mean; Veh, vehicle.

**Figure 8:**
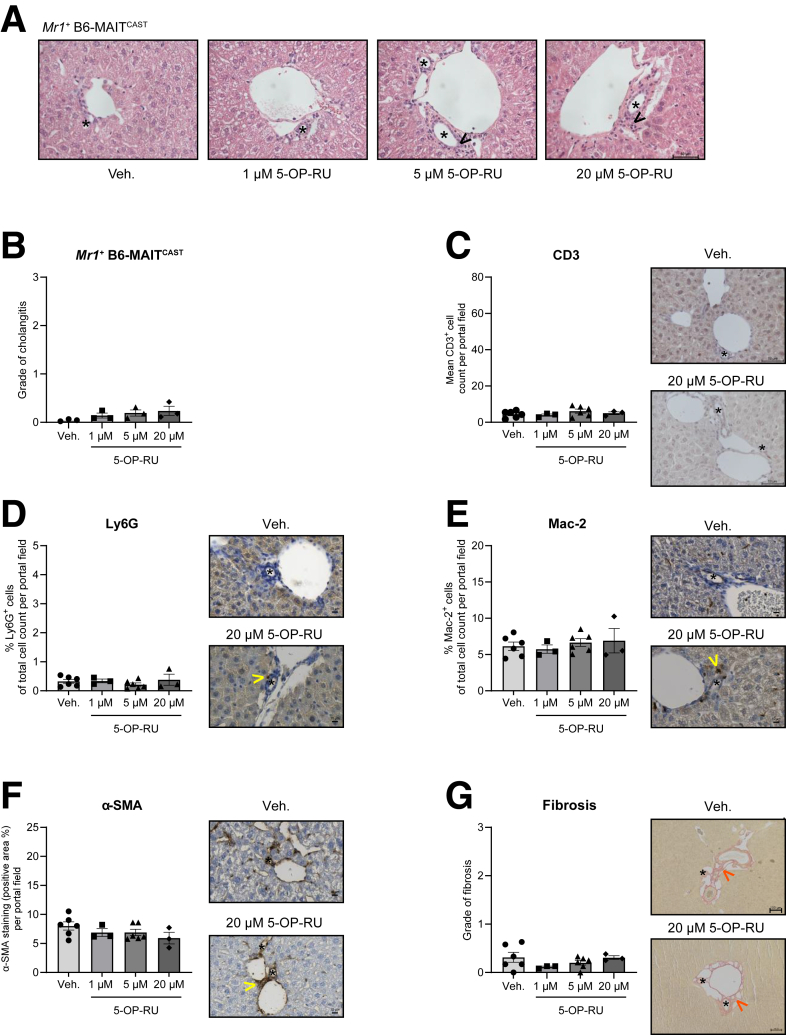
**5-OP-RU induced activation of hepatic MAIT cells does not lead to biliary inflammation in *Mr1*^*+*^ B6-MAIT^CAST^ mice.** (*A*) Representative H&E staining (40×) of liver sections from *Mr1*^*+*^ B6-MAIT^CAST^ mice comparing biliary injection of increasing concentrations of 5-OP-RU with vehicle at day 1. (*B*) Histologic evaluation of cholangitis in *Mr1*^*+*^ B6-MAIT^CAST^ mice following biliary injection of increasing concentrations of 5-OP-RU (n = 3–6) or vehicle (n = 6) at day 1 post surgery. Portal inflammation, necrosis and fibrosis were scored on a 0-3 scale: absent (0), mild (1), moderate (2), and severe (3). (*C–G*) Representative immunohistochemical staining of the inflammatory infiltrate surrounding bile ducts, and corresponding quantification of (*C*) CD3^+^, (*D*) Ly6G^+^, (*E*) Mac2^+^, and (*F*) α-SMA^+^ cells at day 1 after surgery in indicated mice. (*G*) Histologic evaluation of portal fibrosis in the indicated mice, scored as absent (0), mild (1), or moderate (2), with representative Sirius Red-stained liver sections (10×) from day 1 after injection of vehicle or 20 μM 5-OP-RU. Scale bars represent 10 μm (Ly6G/Mac-2/α-SMA), 50 μm (CD3/H&E), and 100 μm (Sirius Red); *asterisks*, bile ducts; *yellow arrowheads*, CD3/Ly6G/Mac-2– or α-SMA–positive cells; *orange arrowheads*, collagen depositions. Each symbol represents a single mouse. Representative results from 1 of 2 independent experiments are shown, presented as mean ± SEM; and (*B–G*) 1-way ANOVA with Dunnett’s adjustment for multiple comparisons test was used to determine statistical differences. SEM, standard error of the mean; Veh, vehicle.

Next, we evaluated whether 5-OP-RU–induced activation of hepatic MAIT cells was paralleled by histopathologic alterations in the liver. The grade of cholangitis at 1 day after surgery showed that intrabiliary injection of the 2 highest doses of 5-OP-RU (5 μmol/L and 20 μmol/L) in iVα19 Cα^−/−^ Tg mice induced significantly increased bile duct inflammation compared with vehicle (Figure 7*A* and *B*). Histologic examination showed an increase in infiltrating T cells at 20 μmol/L 5-OP-RU (Figure 7*C*) accompanied by significant increase in infiltration of neutrophils and macrophages (Figure 7*D* and *E*, respectively) and incipient fibrosis formation as evaluated by α-SMA (Figure 7*F*) and Sirius Red staining at 20 μmol/L (Figure 7*G*). In *Mr1*^*+*^ B6-MAIT^CAST^ mice, despite the activated phenotype observed following biliary injection of 5 μmol/L 5-OP-RU, the MAIT cell frequency was too low to induce biliary inflammation (Figure 8) reflecting that MAIT cells can promote inflammation in MAIT–enriched tissues (Figure 7*A* and *B*) but likely need help from the surroundings when the MAIT cell frequency is low (Figure 8*A–G*).

## Discussion

In this study, we demonstrate that MAIT cell activation by antigens locally presented in the bile ducts, such as microbial derived compounds and the synthetic ligand 5-OP-RU, can drive biliary inflammation and induce tissue damage. This suggests that MAIT cells play a pathogenic role in inflammatory cholangiopathies and therefore potentially represent a novel therapeutic target.

The hepatic immune system is predominantly tolerogenic, but it must also protect against pathogenic microbial infections requiring mechanisms to override immune tolerance.^36^ Given MAIT cells’ localization in close proximity to the bile ducts, which are continuous with the gut lumen and its microbes,^18^^,^^21^^,^^37^ along with recognition of bacterial antigens and rapid proinflammatory cytokine release, they are likely to play an important role in biliary mucosal immune defense in response to bacterial exposure from the gut.^36^^,^^38^^,^^39^ Conditions like dysbiosis can increase the MAIT ligand synthesis^37^ and lead to rapid MAIT cell activation and an inflammatory response.^40^^,^^41^ These reports are of relevance for PSC with the robust clinical association with inflammatory bowel disease along with microbiome alterations.^42^^,^^43^ We have previously reported the presence of MAIT antigens in bile from patients with PSC,^12^ and because cholangiocytes express MR1 and can activate MAIT cells,^21^ these antigens can potentially drive MAIT-cell–mediated bile duct damage in PSC.

We observed that mice with increased MAIT cell frequencies (*Mr1*^*+*^ B6- MAIT^CAST^ and iVα19 Cα^−/−^ Tg mice) exhibited an inflammatory phenotype that was strongest in the iVα19 Cα^−/−^ Tg mice following intrabiliary injection of MAIT cell–activating antigens. This was as expected because iVα19 Cα^−/−^ Tg mice have 30% MAIT cells in the total T cell population compared with 2% in the *Mr1*^*+*^ B6- MAIT^CAST^ mice.^28^^,^^29^ MAIT cells typically function as early responders fighting off infections, but in some circumstances, such as chronic infections, they can modify their function and oppositely contribute to disease pathogenesis.^44^ The underlying molecular mechanisms of this shift is intricate and not well-defined but can be a potential mechanism for a role of MAIT cells in bile duct inflammation with dysregulation and activation by microbial metabolites from bacteria such as *E coli* and *Enterococcus hirae*^45^^,^^46^ impairing MAIT cell function and causing a shift from a protective to a proinflammatory profile.^47^ The potential direct role of bacterial infection in the pathophysiology of cholangiopathies has previously been demonstrated in a PBC model where infection with *Novosphinbium aromaticivorans* promoted disease through activation of *i*NKT cells, another innate-like T cell.^39^^,^^48^ Thus, both direct exposure to antigens from bacterial sources and modified function of MAIT cells can explain their role in cholangitis.

In contrast to the activation in iVα19 Cα^−/−^ Tg mice, the hepatic MAIT and non-MAIT T cells in the *Mr1*^*+*^ B6- MAIT^CAST^ model were similarly activated after injection of fixed *E coli*. With the dramatically lower frequency of MAIT cells in this model, this is not surprising, and other antigens are likely to play a more important role, for instance, through detection by pattern recognition receptors on APCs.^49^^,^^50^ Additionally, MAIT cells can promote bystander activation of other immune subsets like macrophages, neutrophils, and other T cells^41^^,^^51^^,^^52^ in mucosal milieus that are also likely to be relevant in the bile ducts. Thus, in the *Mr1*^*+*^ B6- MAIT^CAST^ model, the net effect of MR1–dependent MAIT cell activation is likely amplified through downstream effector cells.^11^ However, the activation of non-MAIT T cells did not appear to be only secondary to MAIT–driven cholangitis, as MAIT cell–deficient mice (*Mr1*^*−*^ B6-MAIT^CAST^) injected with fixed *E coli* also developed cholangitis.

By using the synthesized antigen 5-OP-RU, we ruled out a role of other antigens, and direct activation of other immune subsets as 5-OP-RU only activate MAIT cells.^53^ In these experiments, we observed a dose-dependent increase in percentage of activated MAIT cells along with reduced hepatic MAIT cell frequency, which is likely due to TCR downregulation, as previously reported.^30^^,^^51^^,^^54^ The small increase in activated hepatic non-MAIT T cells (ie, MR1 tetramer negative) could represent a secondary effect of MAIT cells directing the immune response through crosstalk and recruitment of other immune subsets.^55^ This can either be mediated through inflammatory cytokines such as interferon γ, tumor necrosis factor α, or IL-17 or Toll-like receptor ligands^29^^,^^38^^,^^56^^,^^57^ or the inflammatory milieu generated by activated MAIT cells could augment the presentation of antigens by other APCs in the bile ducts.^16^ In contrast to the nonspecific activation of MAIT cells in *Mr1*^*+*^ B6- MAIT^CAST^ following *E coli* injection, 5-OP-RU injection led to specific MAIT cell activation also in these mice. By performing snRNA-seq of iVα19 Cα^−/−^ Tg mice receiving 5-OP-RU, we observed a transcriptional profile with a shift towards MAIT17 cells and an enrichment of TCR signaling, costimulatory pathways, and effector programs. This points to that biliary antigen exposure induces functional reprogramming of MAIT cells towards a proinflammatory effector state that is capable of driving tissue injury.

It should be noted that the MAIT cell population is more heterogeneous than previously thought, and there are notable developmental and functional differences between transgenic and nontransgenic MAIT cells. In the *i*Vα19 Cα^−/−^ congenic strain, most cells exhibit a naïve phenotype, with about one-third lacking the promyelocytic leukemia zinc finger protein, a hallmark of classical MAIT cells in nontransgenic mice.^28^^,^^29^^,^^58^ Thus, although the *i*Vα19 Cα^−/−^ Tg strain harbors a MAIT cell frequency resembling human conditions, it does not perfectly reflect normal biology.^55^ Although we acknowledge these phenotypic differences, our *Mr1*^*+*^ B6- MAIT^CAST^ data support the findings in the iVα19 Cα^−/−^ Tg model. The use of both these strains gives relevance to the human situation both in terms of the percentage of MAIT cells and their phenotype. Another limitation of our study design using the bile duct injection technique is the inability to perform repeated or continuous injection of compounds, and in future studies, the long-term effects of MAIT cells on bile duct inflammation should be studied using other models and approaches.

In conclusion, we report a pathogenic role of hepatic MAIT cells in a mouse model of bile duct inflammation. These findings are of high relevance to PSC with its associations to the gut microbiota, biliary MAIT cell antigens, and altered MAIT cell frequencies, where the MR1 pathway could represent a link between microbial dysbiosis and immune-mediated liver injury.^16^^,^^24^

## Materials and Methods

### Mice

Specific pathogen-free male and female C57BL/6J mice (The Jackson Laboratory) and previously described *MR1*^*+*^ B6-MAIT^CAST^, *MR1*^*−*^ B6-MAIT^CAST^ and *i*Vα19 Cα^−/−^ Tg transgenic mice, kindly provided by Oliver Lantz,^28^^,^^29^ were used in this study. All animals were housed in a Minimal Disease Unit at the animal facility at Oslo University Hospital Rikshospitalet, Oslo, Norway, in individually ventilated cages (GM500, Techniplast) with a humidity- and temperature-controlled environment on a 12/12-hour day/night cycle with ad libitum access to water and autoclaved rodent chow diet (Labdiet 5021, IPS Products Supplies). All experiments were performed with cohoused, age- and sex-matched mice and with littermate controls for all experiments. Mice undergoing surgery were not fasted and were 9 to 10 weeks of age at the time of surgery, which was carried out during the light cycle. The animal experiments were approved by the Norwegian National Animal Research Authority (project license no. 13409/25769/10499) and carried out in accordance with the European Directive on Protection of animals used for scientific purposes (210/63/EU) and the Norwegian Animal research legislation.

### Preparation of Fixed *E coli*

The *E coli* strain DH5α (18265017, Thermo Fisher Scientific) was cultured overnight at 37 °C in Luria-Bertani broth (Sigma-Aldrich). Live bacteria were counted by the standard plate-counting method, (counts were expressed as CFU/mL), then aliquoted in a solution made by 50% glycerol and 50% fetal calf serum (FCS) and stored at −80 °C. To fixate the *E coli,* aliquots were thawed and washed in PBS, then incubated for 5 minutes at room temperature (RT) in 1% formaldehyde (HistoLab), vortexed, washed, and resuspended in PBS in different dilutions.

### Preparation of 5-OP-RU(5-A-RU/MeG)

5-Amino-6-ribitylamino-2,4(1H,3H)-pyrimidinedione Dihydrochloride (5-A-RU•HCl) (17,014-74-3, MuseChem) was stored at 4 °C in solid form, dissolved in sterile and distilled H_2_O, and frozen at −80 °C in 5 mmol/L stock solutions. Fifteen minutes prior to use, the solid chemical was mixed in 1:1 ratio with methylglyoxal solution (∼40% in H_2_O) (M0252, Sigma-Aldrich) and used at indicated doses.^59^

### The Bile Duct Injection Technique

All procedures were performed as previously described,^14^^,^^27^ using proper analgesia and general anesthesia. In brief, we performed a median laparotomy, clamped the common bile duct, catheterized the gall bladder, and slowly injected 1 μL/g total body weight of fluid into the bile ducts through the catheter, at a maximum speed of 1 μL/s. We injected either fixed *E coli*, 5-OP-RU (both diluted in PBS), or PBS as a vehicle control.

### Mouse Monitoring and Tissue Collection

All mice were monitored as indicated, with daily observation of the clinical state and registration of total body weight. At the selected postoperative endpoints (day 1, day 2, day 7, and day 14), the mice were euthanized by CO_2_ suffocation, and body, liver, and spleen weights were recorded. Degree of pain was evaluated according to a standardized pain assessment form from our local animal facility that was modified to fit our surgery with incorporation of the mouse grimace scale^60^ and with scoring on the following parameters: activity level/behavior (0–3), appearance (0–3), and clinical signs (0–3), where the score indicates degree of pain as either absent (0), mild (1), moderate (2), or severe (3). Trunk blood was obtained by cardiac puncture and incubated 1 hour at RT, followed by centrifugation at 12,000 rpm for 10 minutes at 4 °C. Serum was then collected and stored at −80 °C before analysis of ALT, aspartate transaminase (AST), and alkaline phosphatase using an ADVIA 1800 (Siemens) at The Central Laboratory, Norwegian School of Veterinary Science. For flow cytometry experiments, livers were perfused with 5 mL of cold PBS before livers and spleens were collected in tubes with cold PBS for isolation of primary lymphocytes.

### Extraction of Primary Lymphocytes

To isolate primary lymphocytes for flow cytometry, livers were minced in petri dishes with ice-cold PBS. The suspensions were then filtered through a 70-μm cell strainer, washed with PBS, and pelleted. Liver lymphocytes were isolated using Percoll 40%/70% gradient centrifugation at 700 g for 30 minutes, followed by a final PBS wash. Spleens were homogenized and filtered through a 40-μm cell strainer and centrifugated at 300 to 400 g. The red blood cells were lysed using RBC Lysis Buffer (10X) (Bio-Legend) for 5 minutes. All cell pellets were freshly stained for flow cytometry.

### Flow Cytometry and Antibodies

Primary lymphocytes in single-cell suspensions were incubated with Fc-block (anti-mouse CD16/32, clone 93, BioLegend) in 1:100 dilution for 30 minutes to avoid nonspecific binding. Lymphocytes were stained directly with phycoerythrin-labeled 5-OP-RU-loaded MR1 tetramers (kindly provided by the National Institutes of Health Tetramer Core), and fluorochrome-conjugated monoclonal antibodies (detailed antibody information is provided in Table 1) for 45 minutes at 4 °C in PBS with 2% FCS. Dead cells were excluded using a viability dye (1:100, Zombie NIR, BioLegend). Cells were fixed by 45 minutes incubation in the FoxP3/transcription factor fixation/permeabilization solution (BD Biosciences) and washed and resuspended in flow buffer (PBS supplemented with 2% FCS and 2% antibiotic-antimycotic) before data acquisition. Flow cytometric analysis was performed using BD FACS Verse flow cytometer (BD Biosciences), and results were analyzed in FlowJo software version 10.1 (FlowJo LLC Biosciences).

**Table 1:** Detailed Information on Antibodies Used for FC and IHC

Antibody	Clone	Cat. no	Application	Provided by
Anti-mouse CD16/32	93	101,302	FC	BioLegend
Anti-mouse CD19	6D5	115,530	FC	BD Biosciences
Anti-mouse CD25	PC61	102,016	FC	BioLegend
Anti-mouse CD69	H1.2F3	104,522	FC	BD Biosciences
Anti-mouse TCRβ	H57-597	553,171	FC	BD Biosciences
5-PO-RU-loaded MR1 tetramers	31,650	32,606	FC	National Institutes of Health Tetramer Core
Anti–Mac-2 rat monoclonal	M3/38	CL8942AP	IHC	Cedarlane Labs
Anti–α-SMA rabbit monoclonal	1A4	ab124964	IHC	Abcam
Anti-CD3 rabbit monoclonal	SP7	ab16669	IHC	Abcam
Anti-Ly6G rat monoclonal	1A8	BP0075-1	IHC	BioXCell
Rabbit IgG monoclonal	EPR25A	ab172730	IHC	Abcam
Rat IgG2a κmonoclonal	RTK2758	ab18450	IHC	Abcam
Rat IgG2b κmonoclonal	eB149/10H5	14-4031-82	IHC	eBioScience

α-SMA, α-smooth muscle actin; 5-OP-RU, 5-(2-oxopropylideneamino)-6-d-ribitylaminouracil; CD, cluster of differentiation; FC, flow cytometry; IHC, immunohistochemistry; Ly6G, lymphocyte antigen 6 complex locus G6D; MR1, MHC I-related molecule; TCR, T cell receptor.

### Mucosal-Associated Invariant T Cell Activation Assay

Single-cell suspensions of primary lymphocytes were prepared from *i*Vα19 Cα^−/−^ Tg spleens and cultured in Dulbecco’s Modified Eagle Medium and Glutamax supplemented with 10% FCS (Thermo Fisher Scientific), 1% Gibco antibiotic-antimycotic (10,000 units/mL of penicillin, 10,000 μg/mL of streptomycin, and 25 μg/mL of amphotericin B) (Thermo Fisher Scientific). The cells were maintained in 37 °C incubators with 5% CO_2_ and cultivated for at least 1 hour before start of the experiment. Splenic cells were then plated in 96 round-bottomed plates (5 × 10^5^ cells in 200 μL medium per well, in triplicates) and incubated with either fresh prepared 5-OP-RU (1 nM, 10 nM, 100 nM, 1 μM, or 10 μM) diluted in PBS or PBS, followed by overnight incubation and subsequently subjected to flow cytometric analysis. Primary MAIT cells were identified as TCRβ^+^MR1-5-OP-RU tetramer^+^ cells and activation was assessed by surface expression of CD69 and CD25.

### Histology and Scoring

Standardized set of liver lobes^61^ were formalin-fixed for 18 to 24 hours and paraffin-embedded. Liver tissue was thereafter cut (3-4 μm) and deparaffinized. The hematoxylin and eosin (H&E) staining was performed at the pathology unit at Oslo University Hospital Rikshospitalet, Oslo, Norway. For Sirius red staining, slides were deparaffinized in Histolab-Clear (HistoLab Products), rehydrated, and stained with hematoxylin QS (Vector Laboratories) for 20 seconds. The slides were rinsed in running water and then stained with 0.1% Sirius Red in picric acid for 60 minutes. After a quick dip in 0.5% acetic acid solution, the slides were dehydrated in 100% ethanol, cleared in Histolab-Clear, and mounted.^14^

The liver sections were scored in a blinded fashion as described in,^14^ using the following parameters: Portal inflammation (0–3), fibrosis (0–3), and bile infarcts and necrosis (0–3), where the score indicates degree of pathology as either absent (0), mild (1), moderate (2), or severe (3). Grade of fibrosis was evaluated with H&E, with a score of 0 indicating no fibrosis, 1 indicating expansion of the portal triad by increased fibrotic tissue, 2 indicating porto-portal fibrosis, and 3 indicating septate fibrosis surrounding most lobules. For each mouse, 3 liver lobes were examined, and within each lobe, 8 peribiliary regions were scored individually. The fibrosis score reported for each mouse represents the average scores (first averaged within each lobe, then across the 3 lobes). Images were generated from an Eclipse E400 Microscope with a DS-Fi1 camera controlled by NIS-elements BR 3.1 software (Nikon).^14^

### Immunohistochemistry

Immunohistochemical staining of T-cells (CD3), macrophages (Mac-2), neutrophils (Ly6G), and myofibroblasts (α-SMA) were performed. Detailed antibody information is provided in Table 1. Sections (3–4 μm) of formalin-fixed, paraffin-embedded (FFPE) liver tissue were deparaffinized in Histolab-Clear before antigen retrieval. Rehydrated slides were heated to 95 °C in sodium citrate buffer (10 mmol/L, pH 6) and boiled for 20 minutes, cooled down at RT for 15 minutes, and transferred to PBS. Endogenous peroxidase activity was blocked by incubating in 3% H_2_O_2_ in PBS (0.3% for CD3) for 20 minutes followed by 3 × 5 minutes TBS-T (CD3) or PBS-T (Ly6G/αSMA/Mac-2) wash. For CD3-staining, blocking was performed with 60-minute incubation with Superblock Blocking Buffer (Thermo Fisher Scientific), whereas slides for Ly6G staining were blocked for 30 minutes with Rodent Block M (Biocare Medical), followed by 60-minute blocking with 2.5% ready-to-use normal goat serum (Vector Laboratories). α-SMA and Mac-2 were blocked in 0.5% bovine serum albumin/0.1% Tween 20 (Sigma-Aldrich) for 60 minutes at RT. The slides were incubated at 4 °C overnight with primary antibodies against CD3, α-SMA, Ly6G, and Mac-2. The slides were washed thoroughly in TBS-T (CD3) or PBS-T (Ly6G/αSMA/Mac-2), followed by incubation with secondary antibodies; anti-rabbit (CD3 and α-SMA) or anti-rat (Ly6G and Mac-2) immunoglobulins (Vector Laboratories) for 30 minutes. After 1 more round of washing, the slides were stained with DAB Peroxidase Substrate Kit (Vector Laboratories) for 12 minutes (CD3), 2 minutes (α-SMA/Mac-2), or 1 minute (Ly6G), rinsed in Milli-Q water, and finally counterstained with hematoxylin QS for 13 seconds. After counterstaining, they were rinsed in running water until the water was clear, dehydrated in ethanol, cleared in Histolab-Clear, and mounted.^14^

### Digital Pathology and Whole Slide Image Analysis

High-resolution images of histologic sections stained for Mac-2, Ly6G, and α-SMA were acquired using an automated slide scanner system (Axio Scan Z1, Carl Zeiss Microscopy). Digital analysis of images to estimate the percentage of cells positive to Mac-2, Ly6G, and α-SMA was performed using an open-source machine learning program, QuPath (University of Edinburgh) version 0.5.1. For each liver sample, tissue sections of 3 liver lobes were analyzed. For each lobe, 6 regions (Mac-2 and Ly6G) or 10 regions (α-SMA) with radius 150 μm surrounding bile ducts were selected.

The positive cell detection function in QuPath was used to estimate the percentage of cells positive to Mac-2 and Ly6G, relative to the total count of cells in each selected region. Adjustments for optimized detection and classification of cells were done by visual inspection. The overall percentage of positive cells was calculated as the average of the 18 selected regions for each liver sample.

Digital analysis of α-SMA–positive cells was performed using the pixel classification function in QuPath. Tissue was classified as positive or negative, and the percentage of positive tissue was found relative to the total area of tissue for each selected region. Lumen was classified as ignored area. Adjustments for optimized classification of tissue were done by visual inspection. The overall percentage of positive tissue area was calculated as the average of the 30 selected regions for each liver sample.

### Single-Nucleus RNA Sequencing

Liver sections (3 × 25 μm) from 6 FFPE blocks (n = 3 vehicle controls, n = 3 *i*Vα19 Cα^−/−^ Tg mice injected with 20 μm 5-OP-RU) were processed for snRNA-seq according to manufacturer’s recommendation (“Sample Preparation from FFPE Tissue Sections for GEM-X Flex Gene Expression, CG00078 ǀRev A” (10x Genomics Inc) and subsequently processed for GEM-X Flex gene expression analysis using multiplexing as described in the 10X user guide (“GEM-X Flex Gene Expression Reagent Kits, CG000787ǀRev B”) The default probe set in Chromium Gem x Flex kit lacked probes targeting *Mr1*, and we therefore included custom probes targeting *Mr1*. The custom probes (2.5 μL) were added to the FFPE Hyb Buffer and the 10x Genomics probes to generate the Modified Probe Hybridization Mix, as described in the Technical Note “Custom Probe Design for Visium Spatial Gene Expression and Chromium Single Cell Gene Expression Flex, CG000621ǀRev D.” The individual wash protocol was used, and a total of 80,000 cells were finally added into each reaction (20,000 from each barcode). The number of sample index polymerase chain reaction cycles was set to 9 as indicated from a quantitative polymerase chain reaction run and outlined in the 10X user guide, and the final product was sequenced at a sequencing depth of 40,000 reads per cell to compensate for the volume of cells (4× the minimally recommended from 10X) in 2 NovaSeq X 25B lanes (Illumina, Inc)

### Bioinformatics

The sequenced data was processed with the 10X Cellranger^62^ pipeline using appropriate configurations to deconvolute the unique samples from each sequencing reaction pool and to align the reads to their corresponding probes in the updated probe reference. Using a SCANPY^63^ pipeline, the samples were merged, quality control-filtered, and single-cell variational inference–adjusted.^64^ Principal component analysis plots were generated to adjust for batch variance, and from the same toolkit, we used modules scAR^65^ and Solo^66^ to remove ambient RNA and doublets respectively. Principal component analysis-level sample variation of Uniform Manifold Approximation and Projections was reduced using the harmony algorithm,^67^ and the cell types were annotated using Celltypist^68^ to transfer labels from a previously annotated dataset,^35^ using majority calls to annotate individual clusters. The T/NK cell cluster was subtype classified using *Cd3ε*^−^ filter to identify NK cells, *Trbv19*^+^ to identify MAIT cells, and mean *Infγ*, *Cxcr3* and *Gzmb*-levels relative to mean *Rorc* and *Cxcr6* levels were used to label MAIT1 vs MAIT17 cells. Differentially expressed genes between 5-OP-RU and vehicle MAIT and non-MAIT T cell clusters were identified in SCANPY, using *t* test and appropriate *P* value correction. The Enrichr^31^ web service and the Bioplanet reference database^32^ were used to query pathway activation by feeding in differentially upregulated genes that were significantly regulated after correcting for cell-cycle–related perturbations using cell-cycle scoring and correction in SCANPY.

### Statistical Analysis

All values are presented as mean ± standard error of the mean unless otherwise stated. Statistical significance was evaluated using the unpaired Student’s *t* test for variables meeting criteria of normal distribution and the Mann-Whitney *U* test for variables not meeting the criteria of normal distribution. For experiments where multiple comparisons were included, 1-way analysis of variance (ANOVA), 2-way ANOVA, and 2-way repeated measures ANOVA was used with correction for multiple testing using Dunnett`s, Tukey's, Brown-Forsythe, Welch’s, or Šidák's method. *P* values below .05 were considered statistically significant. For pathway analysis, *P* < .01 was used for significance level for both genes used for the analysis and pathways reported. Statistical tests were performed using the Prism GraphPad software (version 10.0) unless stated otherwise. All authors reviewed and approved the final manuscript.
